# Supplementary material for: SARS-CoV-2 antibody signatures robustly predict diverse antiviral functions relevant for convalescent plasma therapy
Source: medRxiv. 2020 Sep 18:2020.09.16.20196154. Preprint. [Version 1] doi: 10.1101/2020.09.16.20196154 (PMC7523138; doi:10.1101/2020.09.16.20196154)
Supplement: Supplement 2020 [file 97242-2020.09.16.20196154-1.pdf]

**Supplementary Figures**	
Supplementary Figure 1	Plasma and serum IgG isotype and subclass responses across CoV and control antigen types.
Supplementary Figure 2	Plasma and serum IgA, D, and M isotype and subclass responses across CoV and control antigen types.
Supplementary Figure 3	Plasma and serum FcγR binding responses across antibodies specific to CoV and control antigen types.
Supplementary Figure 4	Correlative relationships between antibodies specific to SARS CoV-2 and endemic CoV.
Supplementary Figure 5	Antibody features associated with clinical status
**Supplementary Tables**	
Supplementary Table 1	Demographic data for convalescent plasma (JHMI) and convalescent serum (DHMC)
Supplementary Table 2	Fc Array Reagents
Supplementary Table 3	Sample Dilutions in the Fc Array Assay

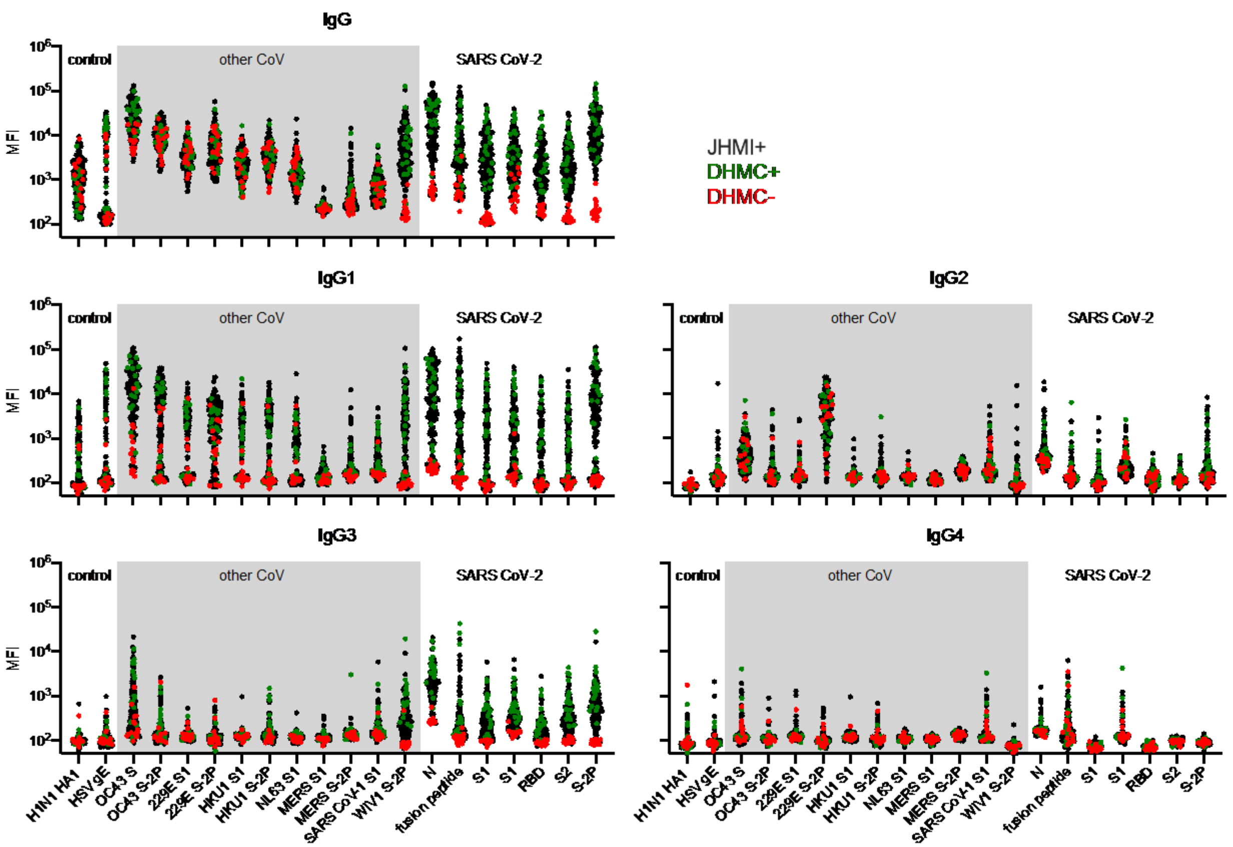

Supplementary Figure 1. Plasma (JHMI) and serum (DHMC) IgG isotype and subclass responses across CoV and control antigens. Samples from convalescent (+) donors are indicated in black and green, and those from SARS-CoV-2 naïve (-) subjects in red.

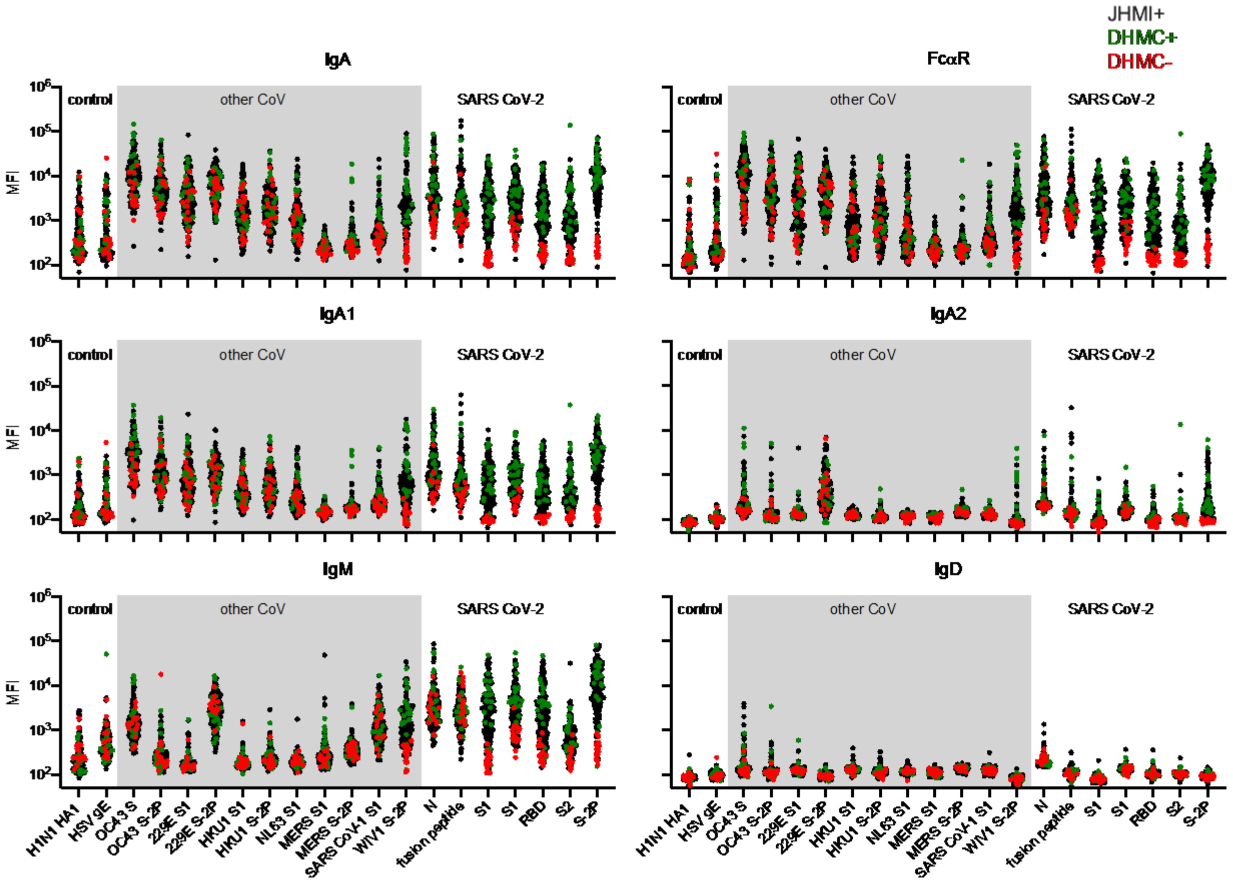

Supplementary Figure 2. Plasma (JHMI) and serum (DHMC) IgA, D, and M isotype and subclass responses across CoV and control antigens. Samples from convalescent (+) donors are indicated in black and green, and those from SARS-CoV-2 naïve (-) subjects in red.

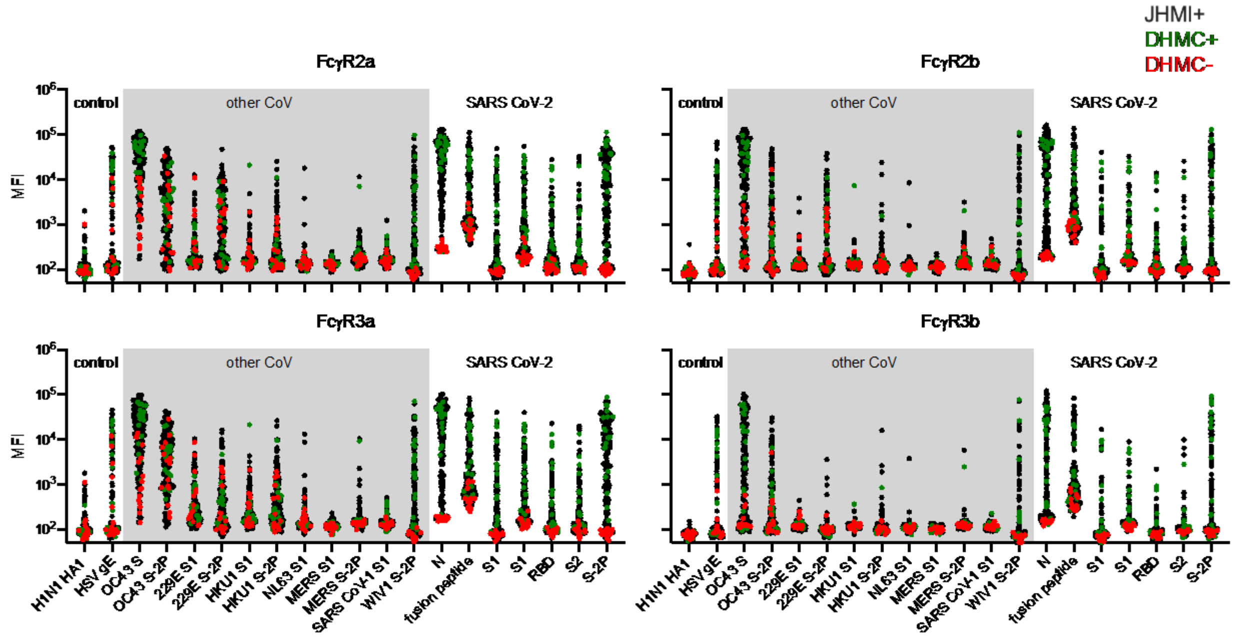

Supplementary Figure 3. Plasma (JHMI) and serum (DHMC) FcγR binding responses across antibodies specific to CoV and control antigens. Samples from convalescent (+) donors are indicated in black and green, and those from SARS-CoV-2 naïve (-) subjects in red.

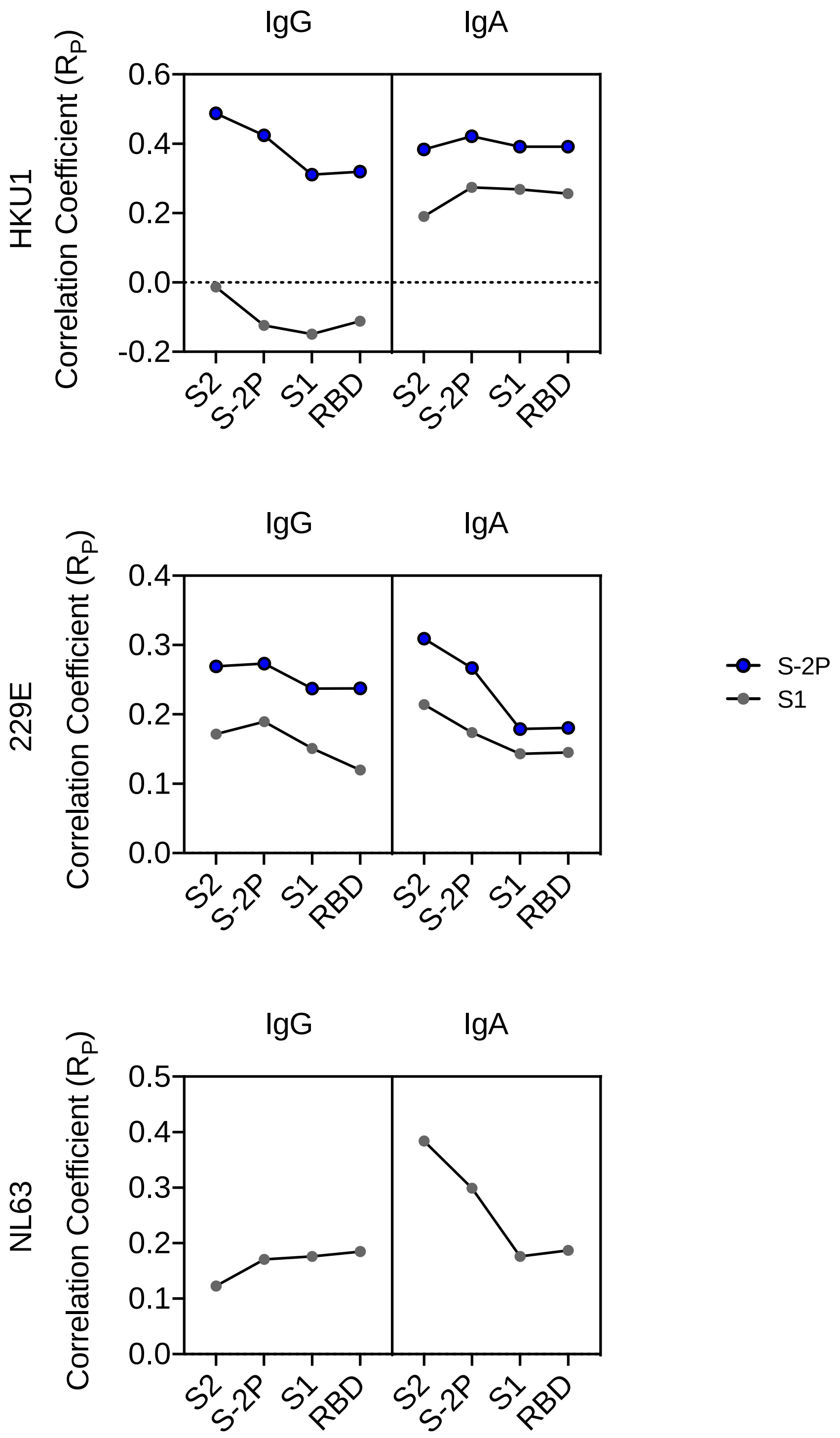

Supplemental Figure 4. Correlative relationships between antibodies specific to SARS-CoV-2 and endemic CoV. Pearson correlation coefficients (Rp) between SARS-CoV-2 specific IgG and IgA responses and IgG and IgA responses to endemic CoV S-2P (blue) and S1 (gray) proteins for HKU1 (top), 229E (center), and NL63 (bottom).

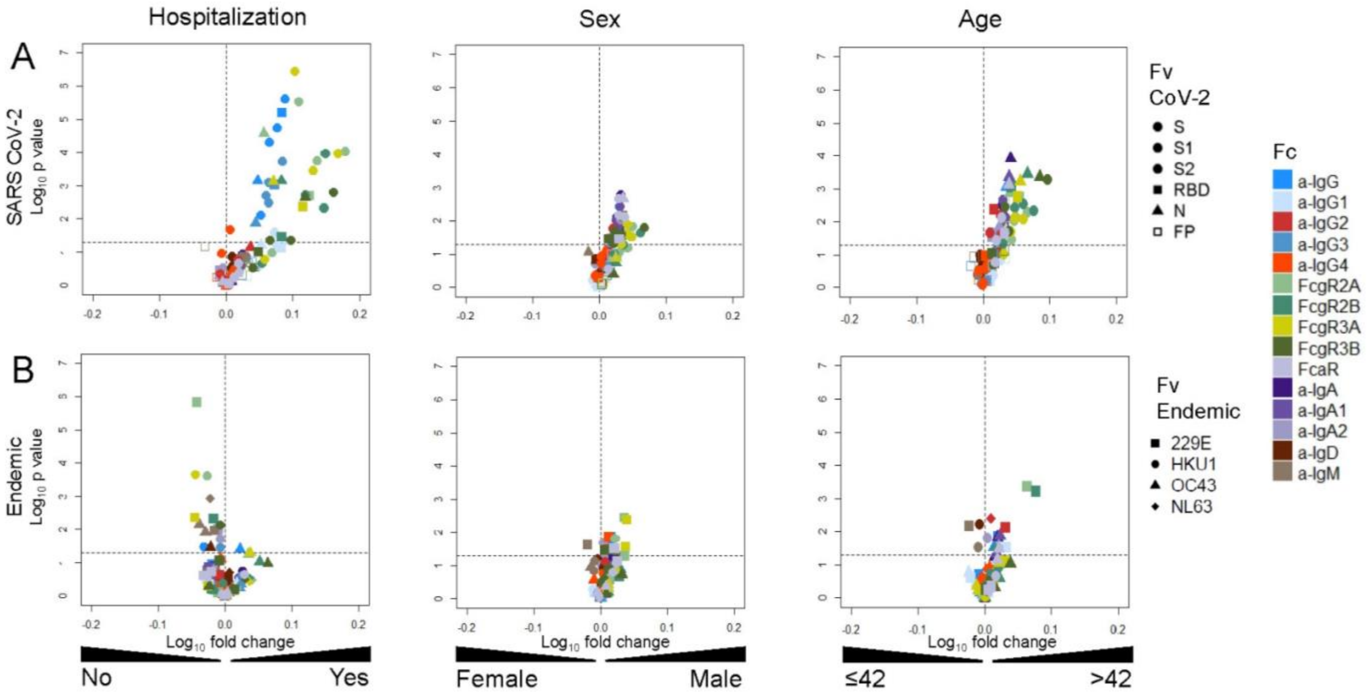

Supplemental Figure 5. Antibody features associated with clinical status. **A-B**. Volcano plots of significance and fold-change of response medians for antibody responses to SARS-CoV-2 (**A**) and endemic CoV (**B**) antigens according to hospitalization status (left), sex (center), and age above or below the cohort median (right). Symbol shapes indicate Fv specificity and color indicates Fc characteristic.

Supplementary Table 1: Demographic information on convalescent plasma (JHMI) and convalescent plasma samples (DHMC).

**Characteristic**	**JHMI Convalescent** n=126	**DHMC Convalescent** n=20	**DHMC Naive** n=15
Median age (IQR), years	42 (29-53)	54 (45-62)	34 (28-52)
Sex			
Female	58 (46%)	10 (50%)	8 (53.3%)
Male	68 (54%)	10 (50%)	7 (46.7%)
Hospitalized (severity)			
No	114 (90.5%)	16 (80%)	NA
Yes	11 (8.7%)	4 (20%)	NA
Unknown	1 (0.8%)		
Median days since PCR+ or symptom onset (IQR)	43 (38-48)	38 (33-45)	NA

Supplementary Table 2: Antigen and Fc Detection Reagents

Antigen	Source	Fc Detection	Source
H1N1 HA1	Immune Technology IT-003-00110p	a- IgG	Southern Biotech 1030-09
HSV gE	Immune Technology IT-005-005p	a-IgG1	Southern Biotech 9054-09
HCoV OC43 S	Sino Biological 40607-V08B	a-IgG2	Southern Biotech 9070-09
OC43 S-2P	Expressed in HEK 293F	a-IgG3	Southern Biotech 9210-09
229E S1	Sino Biological 40605-V08H	a-IgG4	Southern Biotech 9200-09
229E S-2P	Expressed in Expi 293	a-IgA	Southern Biotech 2050-09
HKU1 S1	Sino Biological 40606-V08H	a-IgA1	Southern Biotech 9130-09
HKU S-2P	Expressed in Expi 293	a-IgA2	Southern Biotech 9140-09
NL63 S1	Sino Biological 40604-V08H	a-IgM	Southern Biotech 9020-09
MERS S1	Sino Biological 40069-V08H-50	a-IgD	Southern Biotech 9030-09
MERS S-2P	Expressed in Expi 293	FcαR	Duke Protein Production Facility
SARS CoV-1 S1	Sino Biological 40150-V08B1	FcγR2a	Boesch, et. al, 2014[1]
WIV1 S-2P	Expressed in Expi 293	FcγR2b	Boesch, et. al, 2014[1]
SARS CoV-2 N	Immune Technology IT-002-033Ep	FcγR3a	Boesch, et. al, 2014[1]
SARS CoV-2 fusion peptide	New England Peptide	FcγR3b	Boesch, et. al, 2014[1]
SARS CoV-2 S1 (for Fc Array)	ACROBiosystems S1N-C52H3-100ug		
SARS CoV-2 S1 (for ADCD)	Sino Biological 40150-V08B1-20		
SARS CoV-2 RBD	BEI Resources NR-52366		
SARS CoV-2 S2	Immune Technology IT-002-034p		
SARS CoV-2 S-2P	Expressed in Expi 293		

Supplementary Table 3. Sample Dilutions in the Fc Array Assay

**Sample Type**	**Dilution**	**Detection Reagent**
Serum and Plasma	1:5000	anti-human IgG, FcγR2A, FcγR2B, FcγR3A, FcγR3B
Serum and Plasma	1:1000	anti-human IgG1
Serum and Plasma	1:250	anti-human IgA, anti-human IgA1, anti-human IgA2, anti-human IgD, anti-human IgM, anti-human IgG2 anti-human IgG3, anti-human IgG4, FcαR
